# Supplementary material for: Impact of mental health on burden of illness, septicemia and mortality among patients hospitalized for cancer
Source: PLOS Ment Health. 2024 Nov 25;1(6):e0000005. doi: 10.1371/journal.pmen.0000005 (PMC12798548; doi:10.1371/journal.pmen.0000005)
Supplement: S1 File — (DOCX) [file pmen.0000005.s001.docx]

**S1 File**

**ICD 10 CM billable Codes.**

**Prostate.**

**C61 Malignant neoplasm of prostate**

**(Lung Cancer).**

- **C34.00 Malignant neoplasm of unspecified main bronchus**
- [C34.01](https://www.icd10data.com/ICD10CM/Codes/C00-D49/C30-C39/C34-/C34.01) Malignant neoplasm of right main bronchus
- [C34.02](https://www.icd10data.com/ICD10CM/Codes/C00-D49/C30-C39/C34-/C34.02) Malignant neoplasm of left main bronchus
- [C34.10](https://www.icd10data.com/ICD10CM/Codes/C00-D49/C30-C39/C34-/C34.10) Malignant neoplasm of upper lobe, unspecified bronchus or lung
- [C34.11](https://www.icd10data.com/ICD10CM/Codes/C00-D49/C30-C39/C34-/C34.11) Malignant neoplasm of upper lobe, right bronchus or lung
- [C34.12](https://www.icd10data.com/ICD10CM/Codes/C00-D49/C30-C39/C34-/C34.12) Malignant neoplasm of upper lobe, left bronchus or lung
- [C34.2](https://www.icd10data.com/ICD10CM/Codes/C00-D49/C30-C39/C34-/C34.2) Malignant neoplasm of middle lobe, bronchus or lung
- [C34.30](https://www.icd10data.com/ICD10CM/Codes/C00-D49/C30-C39/C34-/C34.30) Malignant neoplasm of lower lobe, unspecified bronchus or lung
- [C34.31](https://www.icd10data.com/ICD10CM/Codes/C00-D49/C30-C39/C34-/C34.31) Malignant neoplasm of lower lobe, right bronchus or lung
- [C34.32](https://www.icd10data.com/ICD10CM/Codes/C00-D49/C30-C39/C34-/C34.32) Malignant neoplasm of lower lobe, left bronchus or lung
- [C34.80](https://www.icd10data.com/ICD10CM/Codes/C00-D49/C30-C39/C34-/C34.80) Malignant neoplasm of overlapping sites of unspecified bronchus and lung
- [C34.81](https://www.icd10data.com/ICD10CM/Codes/C00-D49/C30-C39/C34-/C34.81) Malignant neoplasm of overlapping sites of right bronchus and lung
- [C34.82](https://www.icd10data.com/ICD10CM/Codes/C00-D49/C30-C39/C34-/C34.82) Malignant neoplasm of overlapping sites of left bronchus and lung
- [C34.90](https://www.icd10data.com/ICD10CM/Codes/C00-D49/C30-C39/C34-/C34.90) Malignant neoplasm of unspecified part of unspecified bronchus or lung
- [C34.91](https://www.icd10data.com/ICD10CM/Codes/C00-D49/C30-C39/C34-/C34.91) Malignant neoplasm of unspecified part of right bronchus or lung
- [C34.92](https://www.icd10data.com/ICD10CM/Codes/C00-D49/C30-C39/C34-/C34.92) Malignant neoplasm of unspecified part of left bronchus or lung

**Malignant neoplasms of lip, oral cavity and pharynx**

(C00-C14).

- - - [C00.0](https://icdlist.com/icd-10/C00.0) - Malignant neoplasm of external upper lip **BILLABLE CODE**
    - [C00.1](https://icdlist.com/icd-10/C00.1) - Malignant neoplasm of external lower lip **BILLABLE CODE**
    - [C00.2](https://icdlist.com/icd-10/C00.2) - Malignant neoplasm of external lip, unspecified **BILLABLE CODE**
    - [C00.3](https://icdlist.com/icd-10/C00.3) - Malignant neoplasm of upper lip, inner aspect **BILLABLE CODE**
    - [C00.4](https://icdlist.com/icd-10/C00.4) - Malignant neoplasm of lower lip, inner aspect **BILLABLE CODE**
    - [C00.5](https://icdlist.com/icd-10/C00.5) - Malignant neoplasm of lip, unspecified, inner aspect **BILLABLE CODE**
    - [C00.6](https://icdlist.com/icd-10/C00.6) - Malignant neoplasm of commissure of lip, unspecified **BILLABLE CODE**
    - [C00.8](https://icdlist.com/icd-10/C00.8) - Malignant neoplasm of overlapping sites of lip **BILLABLE CODE**
    - [C00.9](https://icdlist.com/icd-10/C00.9) - Malignant neoplasm of lip, unspecified **BILLABLE CODE**

[Malignant neoplasm of base of tongue (C01)](https://icdlist.com/icd-10/index/malignant-neoplasm-of-base-of-tongue-c01)

- - - [C01](https://icdlist.com/icd-10/C01) - Malignant neoplasm of base of tongue **BILLABLE CODE**

[Malignant neoplasm of other and unspecified parts of tongue (C02)](https://icdlist.com/icd-10/index/malignant-neoplasm-of-other-and-unspecified-parts-of-tongue-c02)

- - - [C02.0](https://icdlist.com/icd-10/C02.0) - Malignant neoplasm of dorsal surface of tongue **BILLABLE CODE**
    - [C02.1](https://icdlist.com/icd-10/C02.1) - Malignant neoplasm of border of tongue **BILLABLE CODE**
    - [C02.2](https://icdlist.com/icd-10/C02.2) - Malignant neoplasm of ventral surface of tongue **BILLABLE CODE**
    - [C02.3](https://icdlist.com/icd-10/C02.3) - Malig neoplasm of anterior two-thirds of tongue, part unsp **BILLABLE CODE**
    - [C02.4](https://icdlist.com/icd-10/C02.4) - Malignant neoplasm of lingual tonsil **BILLABLE CODE**
    - [C02.8](https://icdlist.com/icd-10/C02.8) - Malignant neoplasm of overlapping sites of tongue **BILLABLE CODE**
    - [C02.9](https://icdlist.com/icd-10/C02.9) - Malignant neoplasm of tongue, unspecified **BILLABLE CODE**

[Malignant neoplasm of gum (C03)](https://icdlist.com/icd-10/index/malignant-neoplasm-of-gum-c03)

- - - [C03.0](https://icdlist.com/icd-10/C03.0) - Malignant neoplasm of upper gum **BILLABLE CODE**
    - [C03.1](https://icdlist.com/icd-10/C03.1) - Malignant neoplasm of lower gum **BILLABLE CODE**
    - [C03.9](https://icdlist.com/icd-10/C03.9) - Malignant neoplasm of gum, unspecified **BILLABLE CODE**

[Malignant neoplasm of floor of mouth (C04)](https://icdlist.com/icd-10/index/malignant-neoplasm-of-floor-of-mouth-c04)

- - - [C04.0](https://icdlist.com/icd-10/C04.0) - Malignant neoplasm of anterior floor of mouth **BILLABLE CODE**
    - [C04.1](https://icdlist.com/icd-10/C04.1) - Malignant neoplasm of lateral floor of mouth **BILLABLE CODE**
    - [C04.8](https://icdlist.com/icd-10/C04.8) - Malignant neoplasm of overlapping sites of floor of mouth **BILLABLE CODE**
    - [C04.9](https://icdlist.com/icd-10/C04.9) - Malignant neoplasm of floor of mouth, unspecified **BILLABLE CODE**

[Malignant neoplasm of palate (C05)](https://icdlist.com/icd-10/index/malignant-neoplasm-of-palate-c05)

- - - [C05.0](https://icdlist.com/icd-10/C05.0) - Malignant neoplasm of hard palate **BILLABLE CODE**
    - [C05.1](https://icdlist.com/icd-10/C05.1) - Malignant neoplasm of soft palate **BILLABLE CODE**
    - [C05.2](https://icdlist.com/icd-10/C05.2) - Malignant neoplasm of uvula **BILLABLE CODE**
    - [C05.8](https://icdlist.com/icd-10/C05.8) - Malignant neoplasm of overlapping sites of palate **BILLABLE CODE**
    - [C05.9](https://icdlist.com/icd-10/C05.9) - Malignant neoplasm of palate, unspecified **BILLABLE CODE**

[Malignant neoplasm of other and unspecified parts of mouth (C06)](https://icdlist.com/icd-10/index/malignant-neoplasm-of-other-and-unspecified-parts-of-mouth-c06)

- - - [C06.0](https://icdlist.com/icd-10/C06.0) - Malignant neoplasm of cheek mucosa **BILLABLE CODE**
    - [C06.1](https://icdlist.com/icd-10/C06.1) - Malignant neoplasm of vestibule of mouth **BILLABLE CODE**
    - [C06.2](https://icdlist.com/icd-10/C06.2) - Malignant neoplasm of retromolar area **BILLABLE CODE**
    - [C06.80](https://icdlist.com/icd-10/C06.80) - Malignant neoplasm of ovrlp sites of unsp parts of mouth **BILLABLE CODE**
    - [C06.89](https://icdlist.com/icd-10/C06.89) - Malignant neoplasm of overlapping sites of oth prt mouth **BILLABLE CODE**
    - [C06.9](https://icdlist.com/icd-10/C06.9) - Malignant neoplasm of mouth, unspecified **BILLABLE CODE**

[Malignant neoplasm of parotid gland (C07)](https://icdlist.com/icd-10/index/malignant-neoplasm-of-parotid-gland-c07)

- - - [C07](https://icdlist.com/icd-10/C07) - Malignant neoplasm of parotid gland **BILLABLE CODE**

[Malignant neoplasm of other and unsp major salivary glands (C08)](https://icdlist.com/icd-10/index/malignant-neoplasm-of-other-and-unsp-major-salivary-glands-c08)

- - - [C08.0](https://icdlist.com/icd-10/C08.0) - Malignant neoplasm of submandibular gland **BILLABLE CODE**
    - [C08.1](https://icdlist.com/icd-10/C08.1) - Malignant neoplasm of sublingual gland **BILLABLE CODE**
    - [C08.9](https://icdlist.com/icd-10/C08.9) - Malignant neoplasm of major salivary gland, unspecified **BILLABLE CODE**

[Malignant neoplasm of tonsil (C09)](https://icdlist.com/icd-10/index/malignant-neoplasm-of-tonsil-c09)

- - - [C09.0](https://icdlist.com/icd-10/C09.0) - Malignant neoplasm of tonsillar fossa **BILLABLE CODE**
    - [C09.1](https://icdlist.com/icd-10/C09.1) - Malig neoplasm of tonsillar pillar (anterior) (posterior) **BILLABLE CODE**
    - [C09.8](https://icdlist.com/icd-10/C09.8) - Malignant neoplasm of overlapping sites of tonsil **BILLABLE CODE**
    - [C09.9](https://icdlist.com/icd-10/C09.9) - Malignant neoplasm of tonsil, unspecified **BILLABLE CODE**

[Malignant neoplasm of oropharynx (C10)](https://icdlist.com/icd-10/index/malignant-neoplasm-of-oropharynx-c10)

- - - [C10.0](https://icdlist.com/icd-10/C10.0) - Malignant neoplasm of vallecula **BILLABLE CODE**
    - [C10.1](https://icdlist.com/icd-10/C10.1) - Malignant neoplasm of anterior surface of epiglottis **BILLABLE CODE**
    - [C10.2](https://icdlist.com/icd-10/C10.2) - Malignant neoplasm of lateral wall of oropharynx **BILLABLE CODE**
    - [C10.3](https://icdlist.com/icd-10/C10.3) - Malignant neoplasm of posterior wall of oropharynx **BILLABLE CODE**
    - [C10.4](https://icdlist.com/icd-10/C10.4) - Malignant neoplasm of branchial cleft **BILLABLE CODE**
    - [C10.8](https://icdlist.com/icd-10/C10.8) - Malignant neoplasm of overlapping sites of oropharynx **BILLABLE CODE**
    - [C10.9](https://icdlist.com/icd-10/C10.9) - Malignant neoplasm of oropharynx, unspecified **BILLABLE CODE**

[Malignant neoplasm of nasopharynx (C11)](https://icdlist.com/icd-10/index/malignant-neoplasm-of-nasopharynx-c11)

- - - [C11.0](https://icdlist.com/icd-10/C11.0) - Malignant neoplasm of superior wall of nasopharynx **BILLABLE CODE**
    - [C11.1](https://icdlist.com/icd-10/C11.1) - Malignant neoplasm of posterior wall of nasopharynx **BILLABLE CODE**
    - [C11.2](https://icdlist.com/icd-10/C11.2) - Malignant neoplasm of lateral wall of nasopharynx **BILLABLE CODE**
    - [C11.3](https://icdlist.com/icd-10/C11.3) - Malignant neoplasm of anterior wall of nasopharynx **BILLABLE CODE**
    - [C11.8](https://icdlist.com/icd-10/C11.8) - Malignant neoplasm of overlapping sites of nasopharynx **BILLABLE CODE**
    - [C11.9](https://icdlist.com/icd-10/C11.9) - Malignant neoplasm of nasopharynx, unspecified **BILLABLE CODE**

[Malignant neoplasm of pyriform sinus (C12)](https://icdlist.com/icd-10/index/malignant-neoplasm-of-pyriform-sinus-c12)

- - - [C12](https://icdlist.com/icd-10/C12) - Malignant neoplasm of pyriform sinus **BILLABLE CODE**

[Malignant neoplasm of hypopharynx (C13)](https://icdlist.com/icd-10/index/malignant-neoplasm-of-hypopharynx-c13)

- - - [C13.0](https://icdlist.com/icd-10/C13.0) - Malignant neoplasm of postcricoid region **BILLABLE CODE**
    - [C13.1](https://icdlist.com/icd-10/C13.1) - Malig neoplasm of aryepiglottic fold, hypopharyngeal aspect **BILLABLE CODE**
    - [C13.2](https://icdlist.com/icd-10/C13.2) - Malignant neoplasm of posterior wall of hypopharynx **BILLABLE CODE**
    - [C13.8](https://icdlist.com/icd-10/C13.8) - Malignant neoplasm of overlapping sites of hypopharynx **BILLABLE CODE**
    - [C13.9](https://icdlist.com/icd-10/C13.9) - Malignant neoplasm of hypopharynx, unspecified **BILLABLE CODE**

[Malig neoplasm of sites in the lip, oral cavity and pharynx (C14)](https://icdlist.com/icd-10/index/malig-neoplasm-of-sites-in-the-lip-oral-cavity-and-pharynx-c14)

- - - [C14.0](https://icdlist.com/icd-10/C14.0) - Malignant neoplasm of pharynx, unspecified **BILLABLE CODE**
    - [C14.2](https://icdlist.com/icd-10/C14.2) - Malignant neoplasm of Waldeyer's ring **BILLABLE CODE**
    - [C14.8](https://icdlist.com/icd-10/C14.8) - Malig neoplm of ovrlp sites of lip, oral cavity and pharynx **BILLABLE CODE**

**Leukemias ICD codes:**

"C9200": **Acute myeloblastic leukemia, not having achieved remission**

"C9201": **Acute myeloblastic leukemia, in remission**

"C9202": **Acute myeloblastic leukemia, in relapse**

"C9210": **Chronic myeloid leukemia, BCR/ABL-positive, not having achieved remission**

"C9211": **Chronic myeloid leukemia, BCR/ABL-positive, in remission**

"C9212": **Chronic myeloid leukemia, BCR/ABL-positive, in relapse**

"C9220": **Atypical chronic myeloid leukemia, BCR/ABL-negative, not achieved remission**

"C9221": **Atypical chronic myeloid leukemia, BCR/ABL-negative, in remission**

"C9222": **Atypical chronic myeloid leukemia, BCR/ABL-negative, in relapse**

"C9300": **Acute monoblastic/monocytic leukemia, not having achieved remission**

"C9310": **Chronic myelomonocytic leukemia not having achieved remission**

"C9311": **Chronic myelomonocytic leukemia, in remission**

"C9312": **Chronic myelomonocytic leukemia, in relapse**

"C93Z0": **Other monocytic leukemia, not having achieved remission**

"C93Z1": **Other monocytic leukemia, in remission**

"C93Z2": **Other monocytic leukemia, in relapse**

"C9390": **Monocytic leukemia, unspecified, not having achieved remission**

"C9391": **Monocytic leukemia, unspecified in remission**

"C9392": **Monocytic leukemia, unspecified in relapse**

"C9400": **Acute erythroid leukemia, not having achieved remission**

"C9401": **Acute erythroid leukemia, in remission**

"C9402": **Acute erythroid leukemia, in relapse**

"C9420": **Acute megakaryoblast leukemia not having achieved remission**

"C9421": **Acute megakaryoblast leukemia, in remission**

"C9422": **Acute megakaryoblast leukemia, in relapse**

"C9430": **Mast cell leukemia not having achieved remission**

"C9431": **Mast cell leukemia, in remission**

"C9432": **Mast cell leukemia, in relapse**

"C9480": **Other specified leukemias not having achieved remission**

"C9481": **Other specified leukemias, in remission**

“C9482”: **Other specified leukemias, in relapse**

**Delirium, dementia, and amnestic and other cognitive disorders**

F0150 F0151 F0280 F0281 F0390 F0391 F04 F05 F070 F0781 F0789 F079 F09 F482 G300 G301 G308 G309 G3101 G3109 G311 G3183 R4181 R54
