# Supplementary material for: Impact of mental health on burden of illness, septicemia and mortality among patients hospitalized for cancer
Source: PLOS Ment Health. 2024 Nov 25;1(6):e0000005. doi: 10.1371/journal.pmen.0000005 (PMC12798548; doi:10.1371/journal.pmen.0000005)
Supplement: Attachment — Submitted filename: Reply2_Revision2_April2024.docx [file pmen.0000005.s002.docx]

**Comments, Journal letter**

PMEN-D-24-00014R1
Impact of Cognitive Disorders On Burden of Illness, Septicemia and Mortality Among Patients Hospitalized for Cancer.
Dr Poolakkad Sankaran Satheeshkumar

Dear Dr. Satheeshkumar,

Thank you for submitting your manuscript entitled "Impact of Cognitive Disorders On Burden of Illness, Septicemia and Mortality Among Patients Hospitalized for Cancer." to PLOS Mental Health. Your manuscript files have been checked in-house, but before we can proceed we need you to address the following issues:


1. We notice that your revision was submitted on March 29, 2024, but the manuscript file in your submission's file inventory was uploaded on January 21, 2024 Please upload the latest version of your revised manuscript as the main article file, with the item type 'Manuscript,' ensuring that it does not contain any tracked changes or highlighting. This will be used in the production process if your manuscript is accepted.

Answers: Thank you for the comments, I have now uploaded the unhighlighted untracked main manuscript as “Manuscript_Main”.

2. We note your current Data Availability statement is:

"We used data from the Publicly available United States’ 2017 National Inpatient Sample database obtained from Healthcare Cost and Utilization Project (HCUP) of the Agency for Healthcare Research and Quality (AHRQ)."

PLOS journals require authors to make all data necessary to replicate their study’s findings publicly available without restriction at the time of publication. When specific legal or ethical restrictions prohibit public sharing of a data set, authors must indicate how others may obtain access to the data. For more information, please see <https://nam12.safelinks.protection.outlook.com/?url=https%3A%2F%2Fjournals.plos.org%2Fplosone%2Fs%2Fdata-availability&data=05%7C02%7Cspoolakk%40buffalo.edu%7Cc4655e97930a43447c9608dc598380d4%7C96464a8af8ed40b199e25f6b50a20250%7C0%7C0%7C638483668538500364%7CUnknown%7CTWFpbGZsb3d8eyJWIjoiMC4wLjAwMDAiLCJQIjoiV2luMzIiLCJBTiI6Ik1haWwiLCJXVCI6Mn0%3D%7C0%7C%7C%7C&sdata=LcxakJQxIx6HUWtHAYlofMoDAgu6BhS8Mx7%2BNXKmg80%3D&reserved=0>.

Before we proceed with your manuscript, please address the following prompts: 

a.) If there are no restrictions, please upload the minimal anonymized data set necessary to replicate your study findings to a stable, public repository and provide us with the relevant URLs, DOIs, or accession numbers. For a list of recommended repositories, please see <https://nam12.safelinks.protection.outlook.com/?url=https%3A%2F%2Fjournals.plos.org%2Fplosone%2Fs%2Frecommended-repositories&data=05%7C02%7Cspoolakk%40buffalo.edu%7Cc4655e97930a43447c9608dc598380d4%7C96464a8af8ed40b199e25f6b50a20250%7C0%7C0%7C638483668538510269%7CUnknown%7CTWFpbGZsb3d8eyJWIjoiMC4wLjAwMDAiLCJQIjoiV2luMzIiLCJBTiI6Ik1haWwiLCJXVCI6Mn0%3D%7C0%7C%7C%7C&sdata=b2xLx2KXQi0KZXxmilutdIsElORd8pz1ofBXViTLm3U%3D&reserved=0>. You also have the option of uploading the data as Supporting Information files, but we would recommend depositing data directly to a data repository if possible.

b.) If there are ethical or legal restrictions on sharing a de-identified data set, please explain them in detail (e.g., data contain potentially identifying or sensitive patient information, data are owned by a third-party organization, etc.) and who has imposed them (e.g., a Research Ethics Committee, Institutional Review Board, third-party data owner, etc.). Please also provide non-author contact information* for a data access committee, ethics committee, or other institutional body to which data requests may be sent.

*PLOS’ Data Policy states that authors cannot be the sole named individuals responsible for ensuring data access.

We will update your Data Availability statement on your behalf to reflect the information you provide.

Answers: Thank you for the comments. I would like to inform you that there are existing usage agreements for the National Inpatient Sample database. Due to these user agreements, I am prohibited from sharing the data with any repository or publication. Conversely, the information is accessible to any anyone from the general public who has an interest in it.

If the journal requires to contact the data distributor, please email:

[**HCUP-RequestData@AHRQ.gov**](mailto:HCUP-RequestData@AHRQ.gov)

Additionally, I am providing I. **summary of the data (Page 2), II. the details about the data user agreement below (Page 3), and III. Purchasing options (Page 8).**

I. **Summary of the National Inpatient Sample (NIS)**

The National Inpatient Sample (NIS) is a component of the Healthcare Cost and Utilization Project (HCUP), which includes a collection of databases and software tools. The NIS is a comprehensive and accessible database that provides information on inpatient healthcare utilization, access, cost, quality, and outcomes at both regional and national levels in the United States. Without considering weight, it encompasses data from approximately 7 million hospital admissions annually. When considering the weight of the data, the estimate is approximately 35 million hospitalizations across the country. The HCUP statistics, which were created as part of a collaboration between the federal government, state governments, and industry, and sponsored by the Agency for Healthcare Research and Quality (AHRQ), are used to make informed decisions at the national, state, and community levels.

Below items are derivative from data user agreements: <https://hcup-us.ahrq.gov/team/NationwideDUA.jsp>

II. **DATA USE AGREEMENT for the Nationwide Databases from the Healthcare Cost and Utilization Project Agency for Healthcare Research and Quality**

This Data Use Agreement ("Agreement") governs the disclosure and use of data in the HCUP Nationwide Databases from the Healthcare Cost and Utilization Project (HCUP) which are maintained by the Agency for Healthcare Research and Quality (AHRQ). The HCUP Nationwide databases include the National (Nationwide) Inpatient Sample (NIS), Kids’ Inpatient Database (KID), Nationwide Ambulatory Surgery Sample (NASS), Nationwide Emergency Department Sample (NEDS), and Nationwide Readmissions Database (NRD). Any person ("the data recipient") seeking permission from AHRQ to access HCUP Nationwide Databases must sign and submit this Agreement to AHRQ or its agent, and complete the online Data Use Agreement Training Course at [www.hcup-us.ahrq.gov](https://hcup-us.ahrq.gov/), as a precondition to the granting of such permission.

Section 944(c) of the Public Health Service Act (42 U.S.C. 299c-3(c)) ("the AHRQ Confidentiality Statute"), requires that data collected by AHRQ that identify individuals or establishments be used only for the purpose for which they were supplied. Pursuant to this Agreement, data released to AHRQ for the HCUP Databases are subject to the data standards and protections established by the Health Insurance Portability and Accountability Act of 1996 (HIPAA) (P.L. 104-191) and implementing regulations ("the Privacy Rule"). Accordingly, HCUP Databases may only be released in "limited data set" form, as that term is defined by the Privacy Rule, 45 C.F.R. § 164.514(e). AHRQ classifies HCUP data as protected health information under the HIPAA Privacy Rule, 45 C.F.R. § 160.103. By executing this Agreement, the data recipient understands and affirms that HCUP data may only be used for the prescribed purposes, and consistent with the following standards:

*No Identification of Persons–* The AHRQ Confidentiality Statute prohibits the use of HCUP data to identify any person (including but not limited to patients, physicians, and other health care providers). The use of HCUP Databases to identify any person constitutes a violation of this Agreement and may constitute a violation of the AHRQ Confidentiality Statute and the HIPAA Privacy Rule. This Agreement prohibits data recipients from releasing, disclosing, publishing, or presenting any individually identifying information obtained under its terms. AHRQ omits from the data set all direct identifiers that are required to be excluded from limited data sets as consistent with the HIPAA Privacy Rule. AHRQ and the data recipient(s) acknowledge that it may be possible for a data recipient, through deliberate technical analysis of the data sets and with outside information, to attempt to ascertain the identity of particular persons. Risk of individual identification of persons is increased when observations (i.e., individual discharge records) in any given cell of tabulated data is ≤10. This Agreement expressly prohibits any attempt to identify individuals, including by the use of vulnerability analysis or penetration testing. In addition, methods that could be used to identify individuals directly or indirectly shall not be disclosed, released, or published. Data recipients shall not attempt to contact individuals for any purpose whatsoever, including verifying information supplied in the data set. Any questions about the data must be referred exclusively to AHRQ. By executing this Agreement, the data recipient understands and agrees that actual and considerable harm will ensue if he or she attempts to identify individuals. The data recipient also understands and agrees that actual and considerable harm will ensue if he or she intentionally or negligently discloses, releases, or publishes information that identifies individuals or can be used to identify individuals.

*Use of Establishment Identifiers–* The AHRQ Confidentiality Statute prohibits the use of HCUP data to identify establishments unless the individual establishment has consented. Permission is obtained from the HCUP data sources (i.e., state data organizations, hospital associations, and data consortia) to use the identification of hospital establishments (when such identification appears in the data sets) for research, analysis, and aggregate statistical reporting. This may include linking institutional information from outside data sets for these purposes. Data recipients are prohibited from identifying establishments directly or by inference in disseminated material. In addition, users of the data are prohibited from contacting establishments for the purpose of verifying information supplied in the data set. Any questions about the data must be referred exclusively to AHRQ. Misuse of identifiable HCUP data about hospitals or any other establishment constitutes a violation of this Agreement and may constitute a violation of the AHRQ Confidentiality Statute.

*Unauthorized Uses of HCUP Data–* Data recipients may only use HCUP data sets for research, analysis and aggregate statistical reporting projects. AHRQ does not authorize the use of HCUP data for commercial or competitive purposes affecting establishments; to determine the rights, benefits, or privileges of individuals or establishments; for criminal and civil litigation, including expert witness testimony; for law enforcement activities; or for any other purpose incompatible with the AHRQ Confidentiality Statute and HCUP data project.

**The undersigned data recipients provide the following assurances concerning HCUP data:**

Protection of Individuals

- I will not release or disclose, and will take all necessary and reasonable precautions to prohibit others from releasing or disclosing, any information that directly or indirectly identifies persons. This includes attempts to identify individuals through the use of vulnerability analysis or penetration testing.
- I acknowledge that the release or disclosure of information where the number of observations (i.e., individual discharge records) in any given cell of tabulated data is ≤10 can increase the risk for identification of persons. I will consider this risk and avoid publication of values of 1-10 in text and tables.
- I will not attempt to link, and will prohibit others from attempting to link, the discharge records of persons in the data set with individually identifiable records from any other source.
- I will not attempt to use and will take all necessary and reasonable precautions to prohibit others from using the data set to contact any persons in the data for any purpose.

Protection of Establishments

- I will not publish or report, through any medium, data that could identify individual establishments directly or by inference.
- When the identities of establishments are not provided in the data sets, I will not attempt to use and will take all necessary and reasonable precautions to prohibit others from using the data set to learn the identity of any establishment.
- I will not contact and will take all necessary and reasonable precautions to prohibit others from contacting establishments identified in the data set to question, verify, or discuss data in the HCUP databases.

Limitations on Data Use, Sharing, and Disclosure

- I will not use or disclose, and I will prohibit others from using or disclosing the data set, or any part thereof, except for research, analysis, and aggregate statistical reporting, and only as permitted by this Agreement.
- I will not use the data set, and I will prohibit others from using the data set, for unauthorized purposes. AHRQ does not authorize the use of HCUP data for commercial or competitive purposes affecting establishments; to determine the rights, benefits, or privileges of individuals or establishments; for criminal and civil litigation, including expert witness testimony; for law enforcement activities; or for any other purpose incompatible with the AHRQ Confidentiality Statute and the HCUP data project. If there is any question regarding whether an intended use of the data set is prohibited, I agree to contact the AHRQ HCUP program for advice prior to engaging in or permitting such use.
- I will not redistribute HCUP data by posting on any website or publishing in any other publicly accessible online repository. If a journal or publication requests access to data or analytic files, I will cite restrictions on data sharing in this Data Use Agreement and direct them to AHRQ HCUP (www.hcup-us.ahrq.gov) for more information on accessing HCUP data.
- I acknowledge that the HCUP NIS, KID, and NRD may contain data elements from proprietary restricted computer software (e.g., 3M™ APR DRGs) supplied by private vendors to AHRQ for the sole purpose of supporting research and analysis with the HCUP NIS, KID, and NRD. While I may freely use these data elements in my research work using the HCUP NIS, KID, and NRD I agree that I will not use and will prohibit others from using these proprietary data elements for any other purpose. In addition, I will enter into a separate agreement with the appropriate organization or firm for the right to use such proprietary data elements for commercial purposes. In particular, I agree not to disassemble, decompile, or otherwise reverseengineer the proprietary software, and I will prohibit others from doing so.

Safeguards

- I will ensure that the data are kept in a secured environment and that only authorized users will have access to the data.
- I will only allow access to HCUP Nationwide data to those who have become authorized users of the HCUP data by signing a copy of this Data Use Agreement and completing the online Data Use Agreement Training Course at www.hcup-us.ahrq.gov. Before granting any individual access to the data set, I will submit the signed data use agreements to the HCUP Central Distributor.

Responsibility

- I acknowledge and affirm that I am personally responsible for compliance with the terms of this Agreement, to the exclusion of any other party, regardless of such party’s role in sponsoring or funding the research that is the subject of this Agreement.
- I acknowledge and affirm that interpretations, conclusions, and/or opinions that I reach as a result of my analyses of the data sets are my interpretations, conclusions, and/or opinions, and do not constitute the findings, policies, or recommendations of the U.S. Government, the U.S. Department of Health and Human Services, or AHRQ.
- I agree to acknowledge in all reports based on these data that the source of the data is the "National Inpatient Sample (NIS), Healthcare Cost and Utilization Project (HCUP), Agency for Healthcare Research and Quality." Substitute "Nationwide Inpatient Sample (NIS)" (if using data prior to 2012), "Kids’ Inpatient Database (KID)," "Nationwide Ambulatory Surgery Sample (NASS)," "Nationwide Emergency Department Sample (NEDS)," or "Nationwide Readmissions Database (NRD)" as appropriate.
- I will indemnify, defend, and hold harmless AHRQ and the data organizations that provide data to AHRQ for HCUP from any or all claims and losses accruing to any person, organizations, or other legal entity as a result of violation of this Agreement. This provision applies only to the extent permitted by Federal and State law.
- I agree to report the violation or apparent violation of any term of this Agreement to AHRQ without unreasonable delay and in no case later than 30 calendar days of becoming aware of the violation or apparent violation.

Terms, Breach, and Compliance

Any violation of the terms of this Agreement shall be grounds for immediate termination of this Agreement. AHRQ shall determine whether a data recipient has violated any term of the Agreement. AHRQ shall determine what actions, if any, are necessary to remedy a violation of this Agreement, and the data recipient(s) shall comply with pertinent instructions from AHRQ. Actions taken by AHRQ may include but not be limited to providing notice of the termination or violation to affected parties and prohibiting data recipient(s) from accessing HCUP data in the future.

In the event AHRQ terminates this Agreement due to a violation, or finds the data recipient(s) to be in violation of this Agreement, AHRQ may direct that the undersigned data recipient(s) immediately return all copies of the HCUP Nationwide Databases to AHRQ or its designee without refund of purchase fees.

Acknowledgment

I understand that this Agreement is requested by the United States Agency for Healthcare Research and Quality to ensure compliance with the AHRQ Confidentiality Statute. My signature indicates that I understand the terms of this Agreement and that I agree to comply with its terms. I understand that a violation of the AHRQ Confidentiality Statute may be subject to a civil penalty of up to $16,443 under 42 U.S.C. 299c-3(d), and that deliberately making a false statement about this or any matter within the jurisdiction of any department or agency of the Federal Government violates 18 U.S.C. § 1001 and is punishable by a fine, up to five years in prison, or both. Violators of this Agreement may also be subject to penalties under state confidentiality statutes that apply to these data for particular states.

Signed:___________________________________________________________________________________________________ Date:__________________________

Print or Type Name:________________________________________________________________________________________________________________________

Organization:________________________________________________________________________________ Title:_________________________________________

Street Address:____________________________________________________________________________________________________________________________

City:_______________________________________________________________________________ State/Province/Region:___________________________________

Postal/ZIP Code:_________________________________________ Country___________________________________________________________________________

Email:_________________________________________________________________________________ Phone:____________________________________________

The information above is maintained by AHRQ only for the purpose of enforcement of this Agreement and for notification in the event data errors occur.

Note to data purchasers: Shipment of the requested data product will only be made to the person who signs this Agreement, unless special arrangements that safeguard the data are made with AHRQ or its agent.

Submission Information

Signed HCUP Data Use Agreements and proof of online training must be submitted to the HCUP Central Distributor for AHRQ’s records. Refer to the Central Distributor page on the HCUP User Support website at <https://hcup-us.ahrq.gov/tech_assist/centdist.jsp> for instructions.

Alternatively, send signed HCUP Data Use Agreements and proof of online training to:

**HCUP Central Distributor**
**E-mail:** [**HCUP-RequestData@AHRQ.gov**](mailto:HCUP-RequestData@AHRQ.gov)
If an alternative option for submission is needed, please contact HCUP Central Distributor at
E-mail: [HCUP-RequestData@AHRQ.gov](mailto:HCUP-RequestData@AHRQ.gov).

Collection of this information is required as a condition of access to Healthcare Cost and Utilization Project data products. The information collection supports requirements of the Public Health Service Act (42 U.S.C. 299c-3(c)), and is used for 1) completion of order transactions, and 2) for enforcement of the HCUP Data Use Agreement. As required by the Privacy Act of 1974, any identifying information obtained will be kept private to the extent provided by law. Public reporting burden for this collection of information is estimated to average 30 minutes per response, the estimated time required to complete the application. An agency may not conduct or sponsor, and a person is not required to respond to, a collection of information unless it displays a currently valid OMB control number. Send comments regarding this burden estimate or any other aspect of this collection of information, including suggestions for reducing this burden, to: AHRQ Reports Clearance Officer Attention: PRA, Paperwork Reduction Project (0935-0206) AHRQ, 5600 Fishers Lane, # 07W41A, Rockville, MD 20857.

OMB Control No. 0935-0206 expires 05/31/2025.

III. Purchasing options (from <https://hcup-us.ahrq.gov/tech_assist/centdist.jsp>)

**HCUP CENTRAL DISTRIBUTOR:** Purchase and Secure Use of HCUP Data

**What is the** [**HCUP Central Distributor**](https://cdors.ahrq.gov)**?**

The [HCUP Central Distributor](https://cdors.ahrq.gov/) Online Reporting System facilitates the HCUP data users with ordering, purchasing, and re-using HCUP databases, including data applications, requesting complementary databases, submitting data re-use and data sharing requests. The website can be found at: <https://cdors.ahrq.gov/> The [HCUP Central Distributor User Guide](https://hcup-us.ahrq.gov/pdf/HCUP_Central_Distributor_User_Guide.pdf) provides detailed instructions on how to purchase, protect, re-use and share the HCUP data.

**How do I Contact the HCUP Central Distributor?**

Email the HCUP Central Distributor Team at: [HCUP-RequestData@ahrq.gov](mailto:HCUP-RequestData@ahrq.gov) (include your order number in subject line, if applicable). Responses may take 5-7 business days.

**What databases can I purchase through the HCUP Central Distributor?**

Nationwide and State databases [available for purchase](https://hcup-us.ahrq.gov/db/availability_public.jsp) are listed on HCUP-US. HCUP Supplemental files including the [American Hospital Association (AHA) linkage files](https://hcup-us.ahrq.gov/db/state/ahalinkage/aha_linkage.jsp), [Cost-to-Charge Ratio files](https://hcup-us.ahrq.gov/db/ccr/costtocharge.jsp) and [Hospital Market Structure files](https://hcup-us.ahrq.gov/toolssoftware/hms/hms.jsp) are available on HCUP-US or through the [HCUP Central Distributor Online Reporting System](https://cdors.ahrq.gov/).

**How do I Purchase HCUP Data?**

1. Register for a User Account on the [HCUP Central Distributor Online Reporting System](https://cdors.ahrq.gov), including submitting the certificate number from required [HCUP DUA Training](https://hcup-us.ahrq.gov/tech_assist/dua.jsp)and the completion date of training.
2. Select the data you wish to purchase from the [HCUP Central Distributor Online Reporting System](https://cdors.ahrq.gov). See [Database Catalog](https://cdors.ahrq.gov/databases). *Nationwide and State database purchases must be in separate orders.* Be sure to also observe the special requirements regarding the purchase of California, Colorado, and Georgia databases, and access to the AHA Linkage and Cost-to-Charge Ratio Files for Iowa, Minnesota, and Nebraska. See [User Guide](https://hcup-us.ahrq.gov/pdf/HCUP_Central_Distributor_User_Guide.pdf).
3. Read and confirm the Data Use Agreements, Responsibility of Data Purchaser, and Indemnification Clause. See Data Use Info & Agreements.
4. For the purchase of State databases, complete the [Statement of Intended Use](https://hcup-us.ahrq.gov/tech_assist/centdist/StatementIntendedUse.pdf) online.
5. Complete Applicant Information as it relates to the specific data purchase, including billing and shipping (for State orders only) information.
6. If applicable, submit [student verification information](https://hcup-us.ahrq.gov/tech_assist/faq.jsp#contactCentDist20).
7. Pay for the databases, once the application is approved. The online system accepts credit card from US banks, ACH, and direct payment methods (wire transfers, purchase orders).
8. Nationwide databases will be available to download from [My Account](https://cdors.ahrq.gov/special-pages/my-account) in the [HCUP Central Distributor Online Reporting System](https://cdors.ahrq.gov). State databases will be shipped on DVDs to you via the United Parcel Service (UPS) (tracked and requiring an adult signature).

NOTE: On occasion, a manual application to purchase data may need to be completed. Please see Applications under Quick References, complete the application, and submit it to [HCUP-RequestData@ahrq.gov](mailto:HCUP-RequestData@ahrq.gov)

**How is the HCUP Data Protected?**

Use of HCUP data requires taking [HCUP Data Use Agreement (DUA) Training](https://hcup-us.ahrq.gov/tech_assist/dua.jsp) and signing an HCUP DUA. Protecting the privacy and security of individuals and institutions and safe handling of the data is critical to the success of HCUP.

- HCUP Data Use Agreement for the Nationwide Databases ([PDF](https://hcup-us.ahrq.gov/team/NationwideDUA.pdf) file, 260 KB; [HTML](https://hcup-us.ahrq.gov/team/NationwideDUA.jsp))
- HCUP Data Use Agreement for the State Databases ([PDF](https://hcup-us.ahrq.gov/team/StateDUA.pdf) file, 251 KB; [HTML](https://hcup-us.ahrq.gov/team/StateDUA.jsp))
- Responsibilities of the HCUP Data Purchaser ([PDF](https://hcup-us.ahrq.gov/tech_assist/centdist/ResponsibilitiesDataPurchaser.pdf) file, 107 KB; [HTML](https://hcup-us.ahrq.gov/tech_assist/centdist/ResponsibilitiesDataPurchaser.jsp))
- Indemnification Clause ([PDF](https://hcup-us.ahrq.gov/tech_assist/centdist/IndemnificationClause.pdf) file, 77 KB; [HTML](https://hcup-us.ahrq.gov/tech_assist/centdist/IndemnificationClause.jsp))

**What, If Any, Requirements Are There Regarding Publishing with HCUP Data?**

Before you publish results based on any HCUP database, confirm that the manuscript follows the requirements of the HCUP Data Use Agreement (DUA). Please review our requirements for publishing with HCUP data [here](https://hcup-us.ahrq.gov/db/publishing.jsp).

**How do I Submit a Data Use Agreement (DUA) without a Purchase?**

If you will be collaborating on another person's data project, or otherwise using HCUP data you do not own, you must submit the appropriate [DUA(s) prior to gaining access to the data](https://cdors.ahrq.gov/data-use-info). To submit a DUA without a data purchase, please complete the [Additional HCUP Data Use Agreements (DUAs)](https://hcup-us.ahrq.gov/pdf/HCUP-NonPurchaser_SubmitDUA.pdf) document and email [HCUP-RequestData@ahrq.gov](mailto:HCUP-RequestData@ahrq.gov). Please include the order number of the original purchase in all correspondence.

**Can I Re-Use the State Data I Purchased for More than One Project?**

Each application and approval for **State-level data** is **project-specific**. If you have State-level HCUP data from a previous project and wish to use the data for a purpose other than that originally approved, or as concrete projects emerge from approved exploratory work, the Data Custodian must submit a "Data Re-Use Request" to the HCUP Central Distributor for review and approval by AHRQ before work may begin on the new project. To submit a data re-use request, please complete the [HCUP State Data Re-Use Request application](https://hcup-us.ahrq.gov/pdf/HCUP-State-Data-Re-use-Application-Kit.pdf) and send it via email to [HCUP-RequestData@ahrq.gov](mailto:HCUP-RequestData@ahrq.gov). Please include the order number of the original data purchase in your email. NOTE: Data Re-Use Requests are not necessary for Nationwide databases.

**How Do I Transfer Custodianship of the Data to Another Person?**

You are the custodian for all data you purchase. If you will be transferring custody to another person, you may request a data custodian transfer by contacting the HCUP Central Distributor. Please remember that individuals, not entities, are responsible for the use and security of the HCUP data, so the data purchaser remains responsible for any use or potential misuse, unless and until custody is formally transferred to another individual. To transfer custody of your data, please complete the [Transfer Custody of HCUP Data application](https://hcup-us.ahrq.gov/pdf/HCUP-Transfer-DataCustodianship.pdf) and send it via an email to [HCUP-RequestData@ahrq.gov](mailto:HCUP-RequestData@ahrq.gov). Please include the order number of the original purchase in your email.
