# Supplementary material for: Impact of mental health on burden of illness, septicemia and mortality among patients hospitalized for cancer
Source: PLOS Ment Health. 2024 Nov 25;1(6):e0000005. doi: 10.1371/journal.pmen.0000005 (PMC12798548; doi:10.1371/journal.pmen.0000005)
Supplement: Attachment — Submitted filename: Reply3_Revision3_Septemb24.docx [file pmen.0000005.s003.docx]

**Point-by-point Response to Reviewers and Editors:**

PMEN-D-24-00014R1
Impact of Cognitive Disorders On Burden of Illness, Septicemia and Mortality Among Patients Hospitalized for Cancer.
PLOS Mental Health

Dear Dr. Satheeshkumar,

Thank you for submitting your manuscript to PLOS Mental Health. I appreciate the improvement made. However, after careful consideration, we feel that it has merit but does not yet fully meet PLOS Mental Health’s publication criteria as it currently stands. Therefore, we invite you to submit a revised version of the manuscript that addresses the points raised during the review process.

==============================
EDITOR:

Your work has a lot of merits. You need to spend a little more time refine it as raised by the reviewers.
==============================

Please submit your revised manuscript by 26th September 2024. If you will need more time than this to complete your revisions, please reply to this message or contact the journal office at mentalhealth@plos.org. When you're ready to submit your revision, log on to https://www.editorialmanager.com/pmen/ and select the 'Submissions Needing Revision' folder to locate your manuscript file.

Please include the following items when submitting your revised manuscript:

- A rebuttal letter that responds to each point raised by the editor and reviewer(s). You should upload this letter as a separate file labeled 'Response to Reviewers'.
- A marked-up copy of your manuscript that highlights changes made to the original version. You should upload this as a separate file labeled 'Revised Manuscript with Track Changes'.
- An unmarked version of your revised paper without tracked changes. You should upload this as a separate file labeled 'Manuscript'.

Guidelines for resubmitting your figure files are available below the reviewer comments at the end of this letter.

We look forward to receiving your revised manuscript.

Kind regards,

Kizito Omona, PhD
Academic Editor
PLOS Mental Health

Journal Requirements:

1. Please review your reference list to ensure that it is complete and correct. If you have cited papers that have been retracted, please include the rationale for doing so in the manuscript text or remove these references and replace them with relevant current references. Any changes to the reference list should be mentioned in the rebuttal letter that accompanies your revised manuscript. If you need to cite a retracted article, indicate the article’s retracted status in the References list and also include a citation and full reference for the retraction notice.

Answers: Reference list has been rechecked, and we confirm that it is complete and correct. And ensure that no cited paper is retracted.

Additional Editor Comments (if provided):

Carefully address the comments raised by the reviewers as fast as you can. Your work has a lot of merit but there is need for some few refinements as noted by the reviewers.

Answers: Thank you for the comments, and we carefully reviewed the comments and transformed the manuscript according to the comments raised by the reviewers.

Reviewers' comments:

Reviewer's Responses to Questions

Comments to the Author

1. If the authors have adequately addressed your comments raised in a previous round of review and you feel that this manuscript is now acceptable for publication, you may indicate that here to bypass the “Comments to the Author” section, enter your conflict of interest statement in the “Confidential to Editor” section, and submit your "Accept" recommendation.

Reviewer #2: All comments have been addressed

Reviewer #3: All comments have been addressed

Reviewer #4: (No Response)

2. Does this manuscript meet PLOS Mental Health’s [publication criteria](https://journals.plos.org/mentalhealth/s/criteria-for-publication)? Is the manuscript technically sound, and do the data support the conclusions? The manuscript must describe methodologically and ethically rigorous research with conclusions that are appropriately drawn based on the data presented.

Reviewer #2: Yes

Reviewer #3: Yes

Reviewer #4: Partly

3. Has the statistical analysis been performed appropriately and rigorously?

Reviewer #2: Yes

Reviewer #3: Yes

Reviewer #4: Yes

4. Have the authors made all data underlying the findings in their manuscript fully available (please refer to the Data Availability Statement at the start of the manuscript PDF file)?

The [PLOS Data policy](https://journals.plos.org/mentalhealth/s/data-availability) requires authors to make all data underlying the findings described in their manuscript fully available without restriction, with rare exception. The data should be provided as part of the manuscript or its supporting information, or deposited to a public repository. For example, in addition to summary statistics, the data points behind means, medians and variance measures should be available. If there are restrictions on publicly sharing data—e.g. participant privacy or use of data from a third party—those must be specified.

Reviewer #2: Yes

Reviewer #3: Yes

Reviewer #4: Yes

5. Is the manuscript presented in an intelligible fashion and written in standard English?

PLOS Mental Health does not copyedit accepted manuscripts, so the language in submitted articles must be clear, correct, and unambiguous. Any typographical or grammatical errors should be corrected at revision, so please note any specific errors here.

Reviewer #2: Yes

Reviewer #3: Yes

Reviewer #4: No

6. Review Comments to the Author

Please use the space provided to explain your answers to the questions above. You may also include additional comments for the author, including concerns about dual publication, research ethics, or publication ethics. (Please upload your review as an attachment if it exceeds 20,000 characters)

Reviewer #2: Good luck

Reviewer #3: The study, "Impact of Cognitive Disorders on Burden of Illness, Septicemia, and Mortality Among Patients Hospitalized for Cancer," presents a well-researched and timely analysis of a critical issue in cancer care. By addressing the intersection of cognitive disorders, septicemia, and mortality, the authors highlight important clinical implications and underscore the need for holistic care approaches for cancer patients. The methodology was robust, and the findings contribute significantly to the literature, offering valuable insights that could inform future healthcare strategies and interventions.

Recommendation:
While the introduction touches on important topics, it lacks clarity in establishing the connection between cognitive disorders, septicemia, and mortality in cancer patients. The rationale behind the study could be further elaborated, as the current narrative does not sufficiently explain the gap in the literature that the research intends to fill. Additionally, the introduction could benefit from a more thorough review of relevant existing studies to better contextualize the study's aims. This would help provide a stronger foundation for the research.

Answers: Thank you for this comment. We have addressed this comment in the introduction.

*Patients undergoing cancer treatment face an increased vulnerability to negative mental health outcomes, particularly those in advanced stages of the disease. These individuals are more susceptible to acute triggers such as infection or the poor consequences of cancer therapy, which can further worsen their mental well-being. [1-3] Furthermore, advanced-stage, old age, or dementia that already exists or is getting worse are added to the complexity. [4,5] Health care providers face difficulties in screening, diagnosing, and monitoring mental illness such as cognitive disorders in cancer care, which cause stress for patients and their caregivers and presents obstacles for medical staff. [6-8] More than one in five U.S. adults experienced mental illness in 2021, totaling 57.8 million people, and one in two may receive a diagnosis at some point in their lives. [11] A wide range of disorders, from mild to severe, fall under the umbrella of "mental illness." Up to one-third of the patients undergoing cancer treatment in hospitals are diagnosed with a prevalent mental health problem. [11, 12] Up to three times as many people in this community suffer from serious mental illnesses than they do in the overall population. [13, 14] Moreover, there is an enormous gap in knowledge when it comes to the impact of the mental health conditions such as cognitive disorders on cancer patients.*

*The published literature focuses on out-patients, with only a few studies including in-patient cohorts. These studies assessed various interventions, such as massage [15], cognitive-behavioral therapy (CBT) [16], collaborative care [17], cost effectiveness [18], coping skills [19], and homebased walking [20]. The outcomes encompassed sleep quality and exhaustion [20], pain and insomnia [21], physical function [22], psychological factors [23], cost [24], and others [25-29]. Nevertheless, research investigating in-hospital outcomes, including mortality, burden of illness, and hospital-acquired complications, is a critical component of patient care that must be taken into account when assessing quality of life. Furthermore, there is a paucity of research examining in-hospital complications on cancer patients who are admitted to hospitals for treatment, indicating lack of research on the outcomes of hospitalized cancer patients with mental illness, resulting in a gap in our present understanding of the burden of illness, problems associated with hospitalization, and mortality rates. The link between these factors have been ascribed to discrepancies in healthcare accessibility encountered by individuals with mental illness. Individuals suffering from psychiatric illness frequently encounter restricted availability of healthcare services, delayed identification of their condition, decreased compliance with treatment, and poor health seeking behaviors. Thus, we hypothesize an association between cancer patients who also suffer from mental illness and unfavorable in-hospital outcomes, including but not limited to burden of illness, in-hospital mortality, and complications associated with the hospital setting.*

*This study focuses the risks and outcomes of mental illness, specifically cognitive disorders, among cancer patients. We aimed to test this hypothesis by analyzing various outcomes, including the burden of illness (such as length of stay and total charge), weight loss, septicemia, fluid and electrolyte imbalance, and in-hospital mortality among cancer patients who were admitted for hospitalization. To conduct this investigation, we utilized the database of the United States' National Inpatient Sample and analyzed patients who were hospitalized for the treatment of leukemia, PC, LC, and OPC. The significance of this study lies in providing valuable information about the impact of current patient care strategies and the impact of mental health on cancer patients.*

Reviewer #4: The manuscript needs to be revised well. I have indicated this specifically in my attached comments. Special attention should be given for the introduction, results, and discussion and the overall write up of the manuscript. With adequate revisions, i hope that the study will be of great value.

Answers: Thank you for this comment, all comments are addressed in the manuscript and indicated below. (Page 6)

7. PLOS authors have the option to publish the peer review history of their article ([what does this mean?](https://journals.plos.org/mentalhealth/s/editorial-and-peer-review-process#loc-peer-review-history)). If published, this will include your full peer review and any attached files.
Answers: Yes, we would like to have publish the peer review history of our article.

Do you want your identity to be public for this peer review? If you choose “no”, your identity will remain anonymous but your review may still be made public.


For information about this choice, including consent withdrawal, please see our [Privacy Policy](https://www.plos.org/privacy-policy).

Reviewer #2: No

Reviewer #3: Yes: Jesan Ara

Reviewer #4: No

[NOTE: If reviewer comments were submitted as an attachment file, they will be attached to this email and accessible via the submission site. Please log into your account, locate the manuscript record, and check for the action link "View Attachments". If this link does not appear, there are no attachment files.]

While revising your submission, please upload your figure files to the Preflight Analysis and Conversion Engine (PACE) digital diagnostic tool, https://pacev2.apexcovantage.com/. PACE helps ensure that figures meet PLOS requirements. To use PACE, you must first register as a user. Registration is free. Then, login and navigate to the UPLOAD tab, where you will find detailed instructions on how to use the tool. If you encounter any issues or have any questions when using PACE, please email PLOS at figures@plos.org. Please note that Supporting Information files do not need this step.

**Comments raised by the reviewer on the manuscript**

1. Please avoid the qualifications from each persons’ name.

Answers: Thank you for the comments, we have now removed the qualification from each person’s name.

2. This has to be stated in the declaration, not here (Funding)

Answers: Thank you for the comments, we have now removed the funding information from the abstract section.

3. The introduction section is not a place to conclude the methods and findings of the study. You must show the scope of the problem, if there are interventions/ guidelines or strategies in place and the aim and significance of the study.

Answers: Thank you for the comments, we have carefully reviewed the comments from reviewer. And removed any methods examining any literature search, and its results from the introduction. And further provided the scope and significance of the study.

And removed the analytical information such as *“We further stratified our analysis into sub-groups”*

Additional grammatical corrections were made.

Changes made in the highlighted section of the manuscript:

*Patients undergoing cancer treatment face an increased vulnerability to negative mental health outcomes, particularly those in advanced stages of the disease. These individuals are more susceptible to acute triggers such as infection or the poor consequences of cancer therapy, which can further worsen their mental well-being. [1-3] Furthermore, advanced-stage, old age, or dementia that already exists or is getting worse are added to the complexity. [4,5] Health care providers face difficulties in screening, diagnosing, and monitoring mental illness such as cognitive disorders in cancer care, which cause stress for patients and their caregivers and presents obstacles for medical staff. [6-8] More than one in five U.S. adults experienced mental illness in 2021, totaling 57.8 million people, and one in two may receive a diagnosis at some point in their lives. [11] A wide range of disorders, from mild to severe, fall under the umbrella of "mental illness." Up to one-third of the patients undergoing cancer treatment in hospitals are diagnosed with a prevalent mental health problem. [11, 12] Up to three times as many people in this community suffer from serious mental illnesses than they do in the overall population. [13, 14] Moreover, there is an enormous gap in knowledge when it comes to the impact of the mental health conditions such as cognitive disorders on cancer patients.*

*The published literature focuses on out-patients, with only a few studies including in-patient cohorts. These studies assessed various interventions, such as massage [15], cognitive-behavioral therapy (CBT) [16], collaborative care [17], cost effectiveness [18], coping skills [19], and homebased walking [20]. The outcomes encompassed sleep quality and exhaustion [20], pain and insomnia [21], physical function [22], psychological factors [23], cost [24], and others [25-29]. Nevertheless, research investigating in-hospital outcomes, including mortality, burden of illness, and hospital-acquired complications, is a critical component of patient care that must be taken into account when assessing quality of life. Furthermore, there is a paucity of research examining in-hospital complications on cancer patients who are admitted to hospitals for treatment, indicating lack of research on the outcomes of hospitalized cancer patients with mental illness, resulting in a gap in our present understanding of the burden of illness, problems associated with hospitalization, and mortality rates. The link between these factors have been ascribed to discrepancies in healthcare accessibility encountered by individuals with mental illness. Individuals suffering from psychiatric illness frequently encounter restricted availability of healthcare services, delayed identification of their condition, decreased compliance with treatment, and poor health seeking behaviors. Thus, we hypothesize an association between cancer patients who also suffer from mental illness and unfavorable in-hospital outcomes, including but not limited to burden of illness, in-hospital mortality, and complications associated with the hospital setting.*

*This study focuses the risks and outcomes of mental illness, specifically cognitive disorders, among cancer patients. We aimed to test this hypothesis by analyzing various outcomes, including the burden of illness (such as length of stay and total charge), weight loss, septicemia, fluid and electrolyte imbalance, and in-hospital mortality among cancer patients who were admitted for hospitalization. To conduct this investigation, we utilized the database of the United States' National Inpatient Sample and analyzed patients who were hospitalized for the treatment of leukemia, PC, LC, and OPC. The significance of this study lies in providing valuable information about the impact of current patient care strategies and the impact of mental health on cancer patients.*

4. The results section should be well organized, well-written and consistent. Please revise it.

Answers: Thank you for the comments, we have now reviewed the results section and provided a well-organized and provided the results in each consistently

Changes made in the highlighted section of the manuscript; additional grammatical corrections were made.

***Prostate cancers***

*Out of the total of 209,410 PCs, there were 16,910 patients who were diagnosed with cognitive disorders. The median age of patients with PC was 70 years, with an interquartile range (IQR) of 63 to 78. Among PC patients with cognitive disorders, the median age was 84 years, with an IQR of 78 to 88. In the unadjusted analysis, patients diagnosed with cognitive disorders showed an extended length of stay (LOS), a greater rate of mortality during their hospital stay, weight loss, imbalances in fluid and electrolyte levels, and septicemia (Table 1). All PC case counts, rates, and estimates are calculated using weighted data.*

*The adjusted analysis showed that PC patients with cognitive disorders were associated with longer LOS (coefficient [coef] = 1.52; 95% confidence interval [CI]: 1.41-1.64). In addition, patients with cognitive disorders were associated with a greater likelihood of developing septicemia (adjusted odds ratio (aOR):1.36; 95% CI :1.22-1.51), weight loss (aOR:1.38; 95% CI: 1.23-1.56), fluid and electrolyte imbalance (aOR: 1.33; 95% CI: 1.21-1.46), and in-hospital mortality (aOR:1.42; 95% CI: 1.21-1.68)Table 5.*

***Lung cancers***

*The study included a total of 403,010 LC cohorts. Among this group, 21,895 patients were diagnosed with cognitive disorders. The median age of patients with LC was 69 years with an IQR of 61 to 76. Among LC patients with cognitive disorders, the median age was 78 years with an IQR of 72 to 84. In the unadjusted analysis, LC patients with cognitive disorders showed extended length of stay (LOS), increased total charges, higher in-hospital mortality rate, and a greater incidence of septicemia in the unadjusted analysis (Table 2). All LC case counts, rates, and estimates are calculated using weighted data.*

*In the adjusted study, LC patients with cognitive disorders were associated with a longer length of stay (coef=1.36, 95%CI: 1.28-1.45) and higher total charges (coef=1.10, 95%CI: 1.05-1.15). Additionally, LC patients with cognitive disorders in the LC group were associated with an increased likelihood of developing septicemia (aOR 1.18; 95% CI: 1.08-1.29), weight loss (aOR 1.53; 95% CI: 1.29-1.53), and fluid and electrolyte imbalance (aOR: 1.16; 95% CI: 1.08-1.25) Table 5.*

***OPC***

*In the study there were 54,264 total number of OPC, among them were 2,275 with cognitive disorders. The median age of the patients with OPC was 63.00 [56.00, 72.00], while among those with OPC and cognitive disorders, it was 77.00 [67.00, 84.25]. In the unadjusted analysis, OPC patients diagnosed with cognitive disorders exhibited increased length of stay (LOS), higher total charges, higher in-hospital mortality rate, and a greater incidence of septicemia (Table 3). All OPC case counts, rates, and estimates are calculated using weighted data.*

*In the adjusted analysis, patients with cognitive disorders in the OPC group were associated with longer length of stay (coef = 1.87, 95% CI: 1.56-2.24), higher total charges (coef = 1.35, 95% CI: 1.17-1.54), and associated with increased odds of developing septicemia (aOR = 1.39, 95% CI: 1.07-1.80) as well as fluid and electrolyte imbalance (aOR = 1.38, 95% CI: 1.10-1.72) Table 5.*

***Leukemia***

*Out of a total of 71,779 patients diagnosed with leukemia, 3050 of them were diagnosed with cognitive disorders. The median age of leukemia patients was 66.00 and IQR of 53.00 and 75.00. Among leukemia patients with cognitive disorders, the median age was 81.00 and IQR of 73.00, 87.00. In the unadjusted analysis, leukemia patients with cognitive disorders were found to have a extended LOS, higher rates of in-hospital mortality, and increased incidence of septicemia (Table 4). All leukemia case counts, rates, and estimates are calculated using weighted data.*

*In the adjusted analysis, leukemia patients who had cognitive disorders were associated with longer LOS (coef = 1.24, 95% CI: 1.03-1.51). Further, leukemia patients who experience cognitive disorders were associated with a greater likelihood of developing septicemia (1.32, 1.07-1.63) and experiencing weight loss (1.34; 1.05-1.69) (Table 5).*

***Sub-group analysis***

*To further, validate our findings where uniform cancer management strategies were adopted or a unique patient sub-population were diagnosed, we chose leukemia patients receiving allogenic hematopoietic stem cell transplant, and LC patients receiving lobectomy treatment. Thus, in the sub-group, we stratified our analysis to those received allogenic hematopoietic stem cell transplant as part of the cancer treatment, and among those treated for cancer of the lung (Malignant neoplasm of LC, Resection of Lung Lobe, Open Approach).*

*Allogenic hematopoietic stem cell transplant (HSCT).*

*There were 6695 (Weighted) Allogenic hematopoietic stem cell transplant patients, and among them there were 135 patients with cognitive disorders. Median [IQR] age of the HSCT patients were 56 [41, 64], and this was 61 [56, 64.25] among patients with cognitive disorders.*

*In the unadjusted analysis, HSCT patients with cognitive disorders were reported to have a higher total charge ($, median [IQR], 635374.38 [430043.56, 950720.19] vs. 405590 [273227.50, 589139.57]; longer LOS (median [IQR], 33 [26.50, 47.50] vs. 26 [22, 31.00]; higher in-hospital mortality (22% vs. 4%); septicemia (44.4% vs. 11.6%); fluid and electrolyte imbalance (85.2% vs. 48.7%).*

*In the adjusted analysis, patients with cognitive disorders were associated with longer LOS, coef: 1.65, 95%CI: 1.26-2.18, and higher total charges, 2.19; 1.36-3.51. Further HSCT patients with cognitive disorders were associated with septicemia, 4.63, 1.86-11.57, and fluid and electrolyte imbalance, 5.02; 1.15-21.9. However, HSCT patients with cognitive disorders were not associated with in-hospital mortality, 3.06, 0.82-11.46.*

*Malignant neoplasm of LC, resection of lung lobe, open approach.*

*There were 8295 (Weighted) malignant neoplasms of lung cancer lobectomy patients, and among them there were 195 patients with cognitive disorders. Median [IQR] age of the malignant neoplasms of LC, lobectomy patients were 67 [59, 73], and this was 72 [69, 77] among patients with cognitive disorders.*

*In the unadjusted analysis, malignant neoplasms of lunger cancer lobectomy patients with cognitive disorders reported longer LOS (median [IQR], 9 [6, 15] vs. 6 [4, 9], weight loss (20.8% vs. 6.4%), fluid and electrolyte imbalance (41.7% vs. 21.9%), higher mortality (12.5% vs. 2.6%), and septicemia (10.4% vs. 3.7%).*

*In the adjusted analysis, patients with cognitive disorders were associated with longer LOS, coefficient: 1.86, 95%CI: 1.33-2.62, and higher total charges: 1.56, 95%CI: 1.06-2.29.*

5. The discussion section must be highly revised. You should start with brief description of the study. For most part, the discussion seems just a literature review. Findings must be adequately compared, and justifications should be provided for discrepancies.

Answers: Thank you for the comments, we have now reviewed the discussion section and provided brief description of the study, and results were adequately compared and justified.

Throughout the manuscript, we have compared out findings to other studies. Changes made in the highlighted section of the manuscript. Additional grammatical corrections were made.

*We found that cancer patients with cognitive disorders has increased LOS, total charges, septicemia, weight loss, and fluid and electrolyte imbalance. Although cancer patients have been conjectured in previous studies concerning the dismal prognosis of mental illnesses—cognitive disorders--, this is the first time it has been reported from a large examination of real-world data from an in-patient hospital record [15-29]. The Diagnostic and Statistical Manual of Mental Disorders (DSM) cognitive disorders—delirium, dementia, amnesia, and other cognitive disorders—were used to categorize mental illnesses (cognitive disorders) in our study. [33] The broad spectrum of cognitive diseases is made up of a diverse group of delirium, dementia, amnesia, and other cognitive disorders where altered cognition caused by recognized disease entities is the fundamental distinguishing feature. [34] Recently, the cognitive disorders were named as neurocognitive disorders with conceptualization of DSM-V. [35, 36] These classifications are intended to promote rigorous clinical reasoning, and the DSM changes are intended to enhance differential diagnostic consideration [37]. As a result, we believe that incorporating delirium, dementia, amnesia, and other cognitive impairments as the illness category could be a useful tool for gauging the cognitive disorder outcome among cancer patients.*

*Research indicate that thirty to fifty percent of cancer patients will have a mental health condition during their cancer journey, according to the evidence that is currently available. Depression, anxiety, and trauma-related disorders are all part of this group. [38] An additional 15–20% of cancer patients also suffer from clinically significant pain, health anxiety, a lack of meaning in life, and other existential issues that are not classified as mental disorders [39]. The prevalence of major depressive disorders in cancer patients is three times higher than the general population, according to estimates [15-29, 40, 41]. In the cancer community, 8–24% of patients report depressive symptoms, and additionally, mental health issues are more common among cancer patients in advanced stages and those receiving palliative care [42, 43]. A high prevalence of mental health difficulties is associated with the emotional and psychological toll that cancer takes on individuals. During the cancer diagnosis, treatment, and aftercare processes, survivors often feel anxious, depressed, and distressed [44]. Since the symptoms of cancer and mental health issues frequently overlap, it can be challenging to detect both and management requires diagnosis and recognition. Evidence suggests that cancer patients with co-occurring mental health disorders are less likely to adhere to their treatment plans, which may result in worse health outcomes [45]. Screening for and providing support for mental health issues is essential to improving the overall health and prognosis of cancer patients. Furthermore, our research reveals that cancer patients who also have cognitive disorders are more likely to experience septicemia, weight loss, and fluid and electrolyte imbalance. This implies the incremental costs burden for cancer patients with cognitive disorders. Due to the prevalence of mental health issues in cancer patients, the impact of these issues can vary depending on their health-seeking behaviors, and sociodemographic factors. As the results remained consistent across all cancer cohorts and the sub-groups, the findings implies the need to screen cancer patients for their met and unmet needs in all stages of their cancer.*

*The prevalence of cancer is increasing world wide at an ever-increasing rate. Survivorship in cancer care and mental health care require attention on mental illness. The impact of cancer upon mental health and impact of management of cancer and mental illness impact the cost of care in hospitalized cancer patients. It is anticipated that almost half of cancer patients may experience some form of mental illness during their journey with the disease. [38] Additionally, cancer diagnosis has significant psychosocial consequences for patients and caregivers; when appropriate interventions were applied, it culminated in better results, as shown by extensive research conducted over the past few decades [46-49]. However, the magnitude of the outcomes are multiple and complex [50], including economic and psychosocial. Recent reports also indicate that mental illnesses are associated with the increased risk of infectious complications following cancer diagnosis [51]. Nonetheless, a patient's health status cannot be accurately measured using a single primary outcome due to the complexity of several mental illnesses and their impact [52]; in addition, patient-defined outcomes, intermediate outcomes, adverse outcomes, and financial consequences are not adequately evaluated most of the time [53]. The research undertaken by Liu, et al [52] investigated the psychological disorders and consequences of septicemia. Furthermore, Lui et al examined the occurrence of pre-cancer mental illness. Unfortunately, we were unable to evaluate this variable due to limitations in our dataset. The results in the hematological patients research differ from ours primarily because our cohort included a large number of patients whereas the Liu, et al, study only included a limited number of individuals who may have received prophylactic antibiotics.*

*It is insufficient to evaluate mental illness solely on one result as opposed to a battery of outcomes. As Grassi, et al captured existential conditions such as demoralization, health anxiety, hopelessness, and existential distress in cancer patients across stages and types of cancer treatment, [54] and further discussed by Caruso, et al, these conditions are not included as 'disorders' in conventional diagnostic and nosological systems [55]. While we explored costs associated as an outcome, consequently, an economic evaluation to detect the effects of mental illness may provide a limited overall estimate. However, the results may be affected by a small proportion, nevertheless, the outcomes could be influenced by a minority group that may depend on healthcare accessibility, racial disparities, and equitable distribution of health resources. [56]. For the last two decades, Quality of Life assessment has been widely used along quantitative measures, Quality-adjusted life-year (QALY) gains––cost per QALY gained––are a metric used in economic evaluations to quantify the health benefit measure utilized to determine the cost of treatments. [57]*

*It should be pointed out that past studies show a 30% economic gap between cancer patients with poor mental health and those with good mental health. [58, 59]. In our study, we found that LC and OPC with cognitive disorders had higher total charges in this study, indicating that the LC and OPCs have a higher associated complication. In addition, in all cancer groups, patients with cognitive disorders had longer LOS. It is important to note that in our study, there is a higher number of patients with cognitive disorders who transferred out to different facilities, and thus the actual cost may be different. According to the most recent Annual Report to the Nation on the Status of Cancer, Part 2, cancer patients in the United States pay a disproportionately high share of cost for cancer care. The national patient economic burden for cancer care in 2019 was $21.09 billion, including $16.22 billion in patient out-of-pocket costs and $4.87 billion in patient time costs [60]. Studies reveal that patients with post-cancer mental diseases suffered the highest expenditures; the greater costs incurred by cancer patients who were later determined to have concurrent mental conditions could be attributed to additional office visits for mental health care as well as an increase in the amount of medication they used [61]. Unfortunately, our data set did not allow examination of the post-cancer treatment options for these patients.*

*Although infectious complications and mental illness are most common among cancer patients, the association between the two is not well understood. Psychological stress, which is recognized to play a major role in the onset, maintenance, and aggravation of mental disorders–as well as in modifying immunological function–is a possible pathway linking mental illness with infections [62, 63]. In this regard, our study showed that among all cancer cohorts, cognitive disorders were associated with the septicemia. This is an area which needs more attention and a more serious implication. Liu et al [52] reported that a statistically significant association between precancer psychiatric disorders and sepsis present between both of their simplified hazard ratios (HR), 1.31; 95% CI, 1.22–1.40, and full HR, 1.26; 95% CI, 1.18–1.35. Additionally noting the findings from a meta-analysis, a pooled relative risk of 2.22 (95% CI, 2.12-2.33) of mortality among people with mental illnesses [63]. Other researchers have also shown a strong association of cognitive disorders and mortality [64-66]. Additionally, our research shows that even after controlling for covariates such as co-morbidities (Elixir Score) in the multivariate analysis, cognitive problems were linked to in-hospital mortality among PC patients. Multiple studies have linked different degrees of cognitive impairment to an increased risk of death [67, 68]. Therefore, it is possible that functional deterioration and underlying co-morbidities are the significant risk factors linked to the aspect's mortality risk.*

*Nonetheless numerous risk factors have been linked to cognitive disorders––old age, poor health, and obesity have been identified as major contributors. [67-71] While it is acknowledged that cognitive disorders are multifactorial maladies caused by the interaction of environmental factors and genetic predisposition, their impact on the aging population with multiple diseases, including cancer, is much greater. [72, 73] In our study, cognitive disorders were associated with fluid and electrolyte imbalance, and weight loss. Reports indicate that people with cognitive disorders, especially those in the early stages were associated with weight loss, which was linked to a decrease in food consumption, and malnutrition [74-76]. On the contrary, being overweight reduced the risk of cognitive disorder related mortality. [77] In other words, the health outcomes of patients with cognitive disorders appear to be poorer for those who are underweight. However, it is not advisable to be either overweight or underweight due to the potential negative health implications.*

*Despite completing their treatment, numerous cancer survivors still grapple with significant mental health issues [38]. Cancer survivors have a greater incidence of mental health disorders, such as depression, anxiety, and psychotic disorders, in comparison to the general population. Individuals who have survived cancer throughout are at a significantly increased risk of getting major depressive disorder, with a fivefold higher likelihood compared to their healthy counterparts [78]. Individuals who have survived colorectal and breast cancer have a higher likelihood, twice as much, of experiencing difficulties with concentration and memory compared to those who have survived other types of cancer [79, 80]. Female cancer survivors more frequently experience cognitive impairments compared to their male counterparts [81]. Further, lower income, social support, and education level are characteristics associated with a higher prevalence of mental health issues among cancer survivors [82]. Enduring cancer can be distressing and disruptive, exacerbating psychological distress. While it is common for cancer survivors to feel anxious and unhappy after treatment, it is important to note that many survivors may still face ongoing mental health challenges [83]. Continuous assistance and supervision are necessary. In order to provide comprehensive care, it is essential to address the mental health concerns of those who have survived cancer. Clinicians should routinely assess survivors for mental health challenges such as anxiety, depression, and cognitive decline, and thereafter connect them with appropriate support services to aid in their coping.*

*Patients who are diagnosed with cancer are more likely to suffer from psychiatric disorders, and the cost of cancer that is accompanied by a mental problem is not proportional to the total cost of both conditions [84]. The research that investigates the connection between cancer and mental illness is still in its infancy, despite the fact that the topic is becoming increasingly important. This is due to the fact that cancer and mental disease are given a disproportionate amount of weight in the profile of healthcare expenditures.*

*Limitations*

*These limitations must be considered when evaluating the findings of the study. First, as with any large database, the quality of data input is primarily determined by those providing the information and is susceptible to variations in reporting and coding. Nonetheless, inconsistencies are believed to be significantly mitigated by the quantity of data included. Secondly, since NIS data includes discharges, it is theoretically possible for a patient to be readmitted and counted more than once, despite the fact that this is exceedingly unlikely, and there are no data on the population of non-admitted patients. Finally, when evaluating resource utilization, we were limited to hospital charges and not actual costs. Even though we controlled potential confounding factors in the study, it is conceivable that there are variables associated with exposure status that we were unable to include in our investigation due to limitations imposed by the dataset. In addition, our analyses only include cancer patients who are hospitalized; hence, the findings may not be applicable to a wider population.*

***Conclusion***

*Cancer patients have a higher risk of having pre-existing mental disorders or developing mental illness, which is more debilitating during cancer recovery––all cancer cohorts in the study demonstrated a significant impact of cognitive disorders, on the burden of illness, weight loss, fluid and electrolyte imbalance, and septicemia. Because of its profound impact on both patients and caregivers, ongoing longitudinal study into cancer survivors' mental health is crucial.*

6. No need to repeat information as this is stated above the abstract

Answers: We have removed the content (correspondence information from the main manuscript, as it is already provided in the title page).
